# Aqueous Levels of Pigment Epithelium-Derived Factor and Macular Choroidal Thickness in High Myopia

**DOI:** 10.1155/2015/731461

**Published:** 2015-09-27

**Authors:** Wei Chen, Yubo Guan, Guanghui He, Zhiwei Li, Hui Song, Shiyong Xie, Quanhong Han

**Affiliations:** ^1^Clinical College of Ophthalmology, Tianjin Medical University, No. 4, Gansu Road, Tianjin 300020, China; ^2^Department of Ophthalmology, Shandong Provincial Hospital Affiliated to Shandong University, Jinan 250000, China

## Abstract

*Purpose*. To investigate the correlation between aqueous and serum levels of pigment epithelium-derived factor (PEDF) and macular choroidal thickness in high myopia patients, both with and without choroidal neovascularization (CNV). *Methods*. Serum and aqueous levels of PEDF were measured by enzyme-linked immunosorbent assay in 36 high myopia patients (36 eyes) with no CNV (non-CNV group), 14 high myopia patients (14 eyes) with CNV (CNV group), and 42 nonmyopia patients (42 eyes) (control group). Macular choroidal thickness was measured by enhanced-depth imaging optical coherence tomography. *Results*. Aqueous levels of PEDF were significantly higher in CNV group compared with non-CNV (*P* < 0.001) and control (*P* < 0.001) groups. Macular choroidal thicknesses were significantly decreased in the non-CNV and CNV groups compared with the control (*P* < 0.001) group. A statistically significant difference (*P* = 0.012) was found between the CNV and non-CNV groups. There was a positive correlation between aqueous PEDF and macular choroidal thickness in the non-CNV group (*P* = 0.005), but no correlation with the CNV group. No correlation between serum PEDF and macular choroidal thickness was detected in the three groups. *Conclusion*. Variations in aqueous PEDF levels coincide with changes in macular choroidal thickness in high myopia patients with no CNV, while no such relationship exists in high myopia patients with CNV.

## 1. Introduction

High myopia, which accounts for 27–33% of all myopia, is a major cause of legal blindness in numerous developed countries worldwide [1], with a prevalence of ~2% in the general population. Pathologically, myopia is characterized by excessive and progressive elongation of the globe (axial length, >26.5 mm) [2] and is associated with degenerative changes of the retina, choroid, and sclera at the posterior segment [3]. Myopic chorioretinal atrophy and choroidal neovascularization (CNV) are common causes of visual loss in high myopes. Since prevention of myopia is presently unachievable, it is of great importance to investigate the underlying mechanisms of and morphological changes associated with chorioretinal atrophy and CNV in highly myopic eyes.

Pigment epithelium-derived factor (PEDF), a secreted 50 kDa glycoprotein belonging to the superfamily of serine protease inhibitors, was first identified in conditioned media of cultured fetal human retinal pigment epithelial (RPE) cells [4]. PEDF not only is a more potent inhibitor of angiogenesis in the eye than are other endogenous antiangiogenic molecules [5], but also has neurotrophic/neuroprotective functions, playing roles in retinal differentiation, survival, and maintenance. Measurable variations in levels of aqueous PEDF in high myopia—with and without CNV—may indirectly reflect the nature and pathogenesis of these two phases of high myopia.

A new technique was recently implemented for improving depth imaging by optical coherence tomography (OCT). This technique—enhanced-depth imaging- (EDI-) OCT—has been shown to produce reliable images of full-thickness choroid [6]. EDI-OCT is, therefore, a most valuable tool for measuring choroidal thickness in highly myopic eyes. Wang et al. reported choroidal thickness to be a better indicator classifying myopic maculopathy than is either axial length or refractive error [7]. Using EDI-OCT, Ohsugi et al. revealed that choroidal thickness in all regions of highly myopic eyes was significantly reduced compared with normal refractive eyes [8].

Whether aqueous levels of PEDF are reflective of stages of chorioretinal atrophy and CNV in high myopia remains to be elucidated. The aim of our study was to determine how changes in choroidal thickness in high myopia contribute to the formation of myopic lesions in CNV. We investigated variations in aqueous and serum levels of PEDF in high myopia patients (with and without CNV) and correlated these variations with macular choroidal thickness. Results are expected to clarify the role of PEDF in the pathophysiology and morphology of high myopia.

## 2. Materials and Methods

### 2.1. Subjects

This observational, comparative, prospective study was carried out at the Tianjin Eye Hospital, Tianjin, China, between August and November 2013. The study conformed to the Declaration of Helsinki tenets for research involving human subjects and was approved by the Institutional Review Board of Tianjin Eye Hospital. Informed consent was obtained from all participants. Patients who had had surgery on both eyes were only enrolled at the time of the first surgery.

Patients were divided into groups based on myopia with and without CNV. The* non-CNV group* comprised 36 high myopia patients (*n* = 36 eyes) (with no CNV) who were in need of cataract surgery. Inclusion criteria included patients with eyes of axial lengths of ≥26.5 mm (Miller and Singerman, 2001) [9], refractive errors of >6 diopters (D), and no apparent macular abnormalities (e.g., choroidal neovascularization, macular holes) and who were 50–70 years of age. Exclusion criteria included poor image quality on OCT as a result of unstable fixation, severe cataract, previous ocular surgery, use of immunosuppressive drugs, eye diseases (e.g., glaucoma, age-related macular degeneration, and retinal detachment), and systemic diseases like serious heart, lung, liver, or kidney dysfunction. Patients with eyes in which the chorioscleral interface could not be clearly visualized were also excluded. The* CNV group* comprised 14 high myopia patients (*n* = 14 eyes) who were in need of intravitreal injections of ranibizumab (Lucentis, Novartis, Switzerland). Inclusion criteria comprised patients with eyes of axial lengths of ≥26.5 mm, refractive errors of >6 D, and choroidal neovascularization by fundus fluorescein angiography (FFA) and who were 50–70 years of age. Exclusion criteria were the same as those for the non-CNV group. Patients with secondary choroidal neovascular diseases, for example, angioid streaks and ocular trauma, were also excluded. All cases of CNV were confirmed by FFA. The* control group* comprised 42 normal patients (*n* = 42 eyes) who were in need of cataract surgery. Inclusion criteria comprised patients with healthy eyes with spherical equivalents of −3D–+3D. Exclusion criteria were the same as for the other groups.

### 2.2. Aqueous and Serum Samples

Samples of undiluted aqueous humor (100–200 *μ*L) were collected by aspiration into a 1 mL syringe at the start of cataract surgery or before a single 0.05 mL intravitreal injection of ranibizumab. Serum specimens were collected prior to surgery. The levels of PEDF in aqueous humor and serum were measured using enzyme-linked immunosorbent assay (ELISA), according to the manufacturer's instructions (ChemiKine, Temecula, California, USA).

### 2.3. Ophthalmic Examination

All patients underwent a complete ophthalmic examination, including assessment of visual acuity (VA), refractive error, intraocular pressure (IOP), and axial length; OCT; dilated fundus examination by indirect ophthalmoscopy; and color fundus photographic assessment of myopic maculopathy. FFA was performed on all high myopia patients to confirm the presence or absence of CNV.

### 2.4. Measurements

#### 2.4.1. Choroidal Thickness

**Figure 1:**
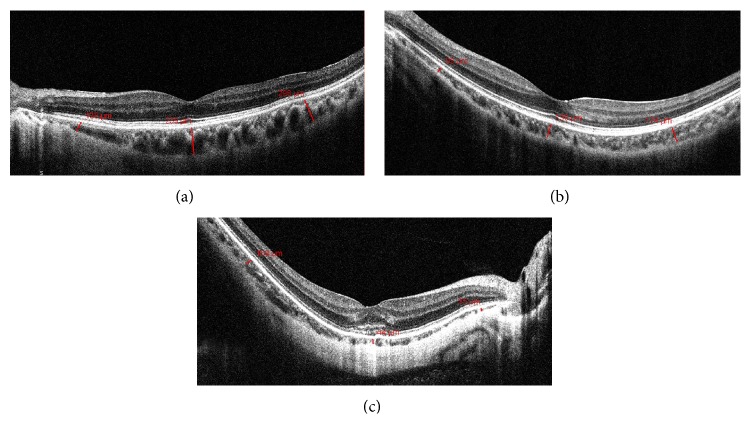
Optical coherence tomography (OCT) images using enhanced-depth imaging. The choroidal thickness (red line) is defined as the vertical from the outer surface of the hyperreflective line ascribed to the retinal pigment epithelium (RPE) to the hyperreflective line of the inner sclera border (a). Representative scan of a control individual (b). Representative scan of an individual with high myopia without choroidal neovascularization (CNV); note that the choroid is thinner than in the control but thicker than in high myopia with CNV (c). Representative scan of an individual with high myopia with CNV; note that the choroid is thinner than that of control or high myopia without CNV.

The scan protocol of the Cirrus OCT (Carl Zeiss Meditec, Jena, Germany) generates a cube of data through a 9 mm line consisting of 4,096 A-scans around the macular region via a HD 5-line raster mode. EDI-OCT protocols have been described elsewhere [10]. Using the software-provided caliper system, choroidal thickness was measured from the outer surface of the hyperreflective line ascribed to the retinal pigment epithelium (RPE) to the hyperreflective line of the inner scleral border (Figure 1). Choroidal thicknesses were measured at the fovea, 3 mm superior and inferior to the fovea in vertical sections, and 3 mm temporal and nasal to the fovea in horizontal sections. The mean overall choroidal thickness, recorded as macular choroidal thickness, was obtained by calculating average choroidal thickness measurements from all measured areas. Two independent observers manually measured each choroidal thickness; both sets of measurements were averaged for analysis.

#### 2.4.2. Axial Length, Refractive Error, and IOP

Axial length was measured using the Intraocular Lens Master (IOL-Master; Carl Zeiss Meditec, Dublin, CA). Refractive error was measured by autorefractometry (RK-3; Canon, Tokyo, Japan). IOP was measured by noncontact tonometry (TX-20 model; Canon, Tokyo, Japan).

### 2.5. Statistical Analysis

Statistical analyses were performed using version 17.0 SPSS software (SPSS, Inc., Chicago, IL, USA). All data were described as mean ± standard deviation (SD), with a 95% confidence interval (CI). An unpaired* t*-test was used to compare two independent groups with normal distribution; the Mann-Whitney* U* test was used to compare two independent groups without normal distribution; the Kruskal-Wallis* H*-test was used to compare variables among different groups; Fisher's exact* t*-test was used to compare noncontinuous variables; and Pearson's correlation test was used to analyze the correlation between aqueous PEDF concentrations, macular choroidal thickness, and serum PEDF concentrations for the three groups. A *P* < 0.05 was considered statistically significant.

## 3. Results

Table 1 summarizes demographic and clinical characteristics. Average axial globe lengths were 24.3 ± 0.6, 28.5 ± 1.1, and 29.6 ± 1.3 mm for the control, non-CNV, and CNV groups, respectively. Average refractive errors (spherical equivalent refraction) were −0.29 ± 1.42, −12.3 ± 4.7, and −15.2 ± 3.1 D for the control, non-CNV, and CNV groups, respectively. Significant differences among the three groups were seen in axial length and refractive error (*P* < 0.001). Diffuse chorioretinal atrophy was present in 28 of 36 (77.8%) highly myopic eyes in the non-CNV group.

**Figure 2:**
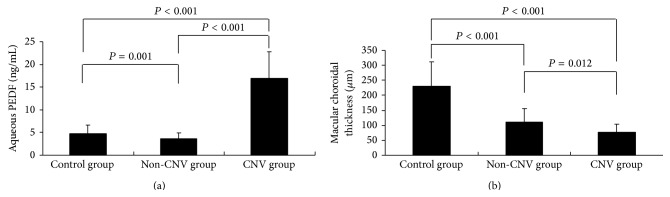
Aqueous pigment epithelium-derived factor (PEDF) levels in control and non-CNV groups (a) and in high myopia with CNV group. (b) Macular choroidal thickness in control, non-CNV, and CNV groups. Results are geometric mean (95% CI).

Aqueous levels of PEDF were significantly decreased in the non-CNV group (3.6 ± 1.3 ng/mL) compared with the control group (4.8 ± 1.8 ng/mL) (*P* = 0.001) and were significantly elevated in the CNV group (17.0 ± 5.8 ng/mL) compared with the other two groups (*P* < 0.001) (Figure 2). Mean serum concentrations of PEDF were 5.8 ± 1.3, 5.4 ± 1.2, and 6.1 ± 1.5 *μ*g/mL in the control, non-CNV, and CNV groups, respectively, with no statistically significant differences (*P* = 0.632). Aqueous levels of PEDF were significantly lower than serum levels of PEDF in all three groups (*P* < 0.001). Mean macular choroidal thicknesses were 230.6 ± 81.8, 111.1 ± 45.0, and 77.2 ± 26.9 *μ*m in the control, non-CNV, and CNV groups, respectively. Differences were statistically significant for the non-CNV and CNV groups compared with the control group (*P* < 0.001), while a significant difference (*P* = 0.012) was also found between the non-CNV and CNV groups (Figure 2).

**Figure 3:**
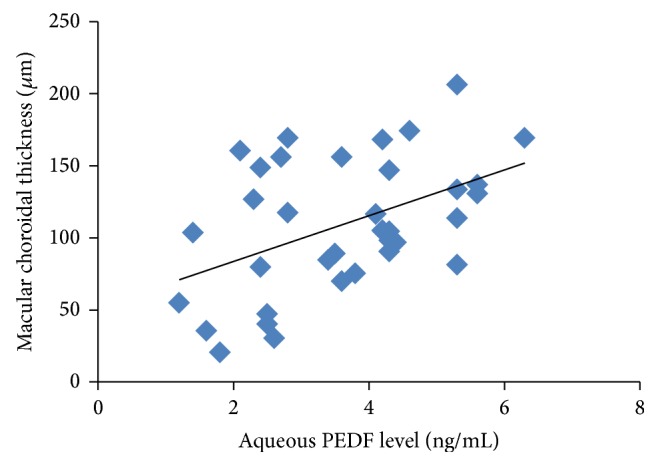
Scatterplots of aqueous PEDF levels and macular choroidal thickness in the non-CNV group show a significant positive correlation (*R* ^2^ = 0.211;* y* = 15.852*x* + 51.986; *P* = 0.005).

**Figure 4:**
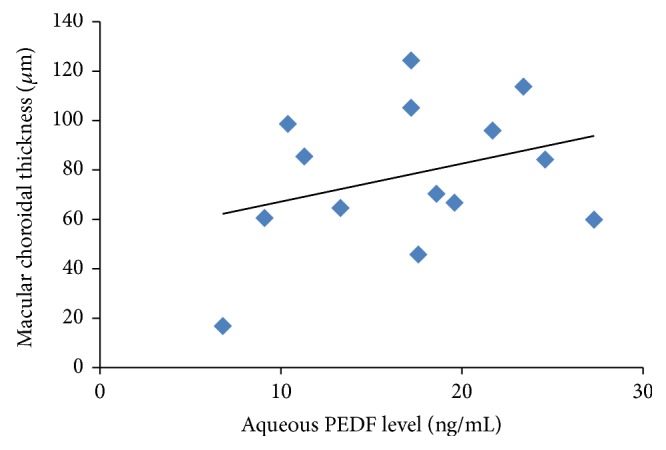
Scatterplots of aqueous PEDF levels and macular choroidal thickness in the CNV group show no correlation (*R* ^2^ = 0.108;* y* = 1.5162*x* + 51.404; *P* = 0.214).

We studied the correlation between aqueous PEDF levels and macular choroidal thickness for the non-CNV group and found a significant, positive correlation (*R* ^2^ = 0.211, *P* = 0.005) (Figure 3), while no correlation was found for the CNV group (*R* ^2^ = 0.108, *P* = 0.214) (Figure 4). Conversely, no correlation between serum PEDF and macular choroidal thickness was detected for the non-CNV and CNV groups (*R* ^2^ = 0.078, *P* = 0.674;* R* ^2^ = 0.0064, *P* = 0.720, resp.). Also no correlation was found between aqueous and serum concentrations of PEDF for the non-CNV and CNV groups (*R* ^2^ = 0.017 and* R* ^2^ = 0.0054, resp.). There were also no correlations between aqueous PEDF, serum PEDF, and macular choroidal thickness for the control group.

## 4. Discussion

Pathologic myopia with progressive and excessive elongation of the eyeball results in a number of chorioretinal changes, including posterior staphyloma, chorioretinal atrophy, and pathologic CNV. Although the mechanisms underlying the development of chorioretinal atrophy and CNV remain unknown, dysfunctional RPE and RPE retraction—in the direction of radial traction line from its underlying glial tissue—are important contributors [11].

PEDF is synthesized and secreted by RPE and retinal ganglion cells and diffuses into the vitreous and aqueous humors [12]. In high myopia, the mechanical tissue strain caused by axial elongation could lead to the development of choroidal ischemia, followed by RPE atrophy. There is a trend toward fewer photoreceptor cells and decreased RPE cell density in eyes with pathological myopia [13]. In addition, age-related functional deterioration of the RPE could result in altered PEDF expression, consequently decreasing inhibition of CNV growth [14].

In the present study, we found that levels of aqueous PEDF in non-CNV eyes were significantly lower (3.6 ± 1.3 ng/mL) than in the control group (*P* = 0.001). Ogata et al. also found the mean aqueous PEDF levels in high myopia to be significantly lower than in eyes with cataract alone [15]. However, Shin et al. reported that PEDF concentrations in highly myopic eyes did not differ significantly from those of control eyes [16]. We speculate that there are two reasons for the decrease found in the non-CNV group. First, because of the elongated axial length of highly myopic eyes, PEDF concentrations may be diluted as a result of the larger vitreous cavity, leading to decreased aqueous concentrations. Second, with chorioretinal atrophy-associated high myopia, decreased PEDF production may be a consequence of degenerated RPE and retinal ganglion cells, its two main sources in the eye.

Vascular endothelial growth factor (VEGF) and PEGF are two major cytokines in angiogenesis. VEGF not only is the major stimulator of neovascular growth and vascular permeability, but also maintains normal functions in many normal adult tissues. The aqueous level of VEGF has been analyzed in high myopia in another study of our group. We found aqueous levels of VEGF from non-CNV high myopia patients were significantly lower compared with those from control persons (*P* < 0.001); meanwhile aqueous levels of VEGF were significantly associated with both macular choroidal thickness (*P* < 0.001) and axial length (*P* < 0.001) [17]. Interestingly, in a similar study, Shin et al. reported that aqueous levels of VEGF in high myopia without CNV were significantly lower than levels in normal eyes [16].

Aqueous levels of PEDF were significantly increased in the CNV group compared with the non-CNV and control groups (*P* < 0.001). Ogata et al. demonstrated increased expression of both VEGF and PEDF in retinas with experimentally induced CNV [18]. However, Holekamp et al. demonstrated lower PEDF, but not VEGF, levels in the vitreous of patients with active CNV resulting from age-related macular degeneration (AMD) [19].

In the present study, we found no significant differences in serum PEDF levels among groups. Furthermore, there were no significant correlations between concentrations of aqueous and serum PEDF for the non-CNV and CNV groups. These findings suggest that, in high myopia, aqueous concentrations of PEDF are not determined by serum concentrations but, rather, by alterations in intraocular synthesis of PEDF.

In our study, macular choroidal thicknesses were 111.1 ± 45.0 and 77.2 ± 26.9 *μ*m in the non-CNV and CNV groups, respectively, both significantly thinner than that of the control group (*P* < 0.001). In addition, Ikuno et al. reported that eyes with myopic CNV have a thinner choroid in comparison to the contralateral eye without CNV [20]. Chung et al. also speculated that a thinner choroid is a risk factor for CNV in eyes with high myopia [21]. We found that choroidal thickness differed significantly (*P* = 0.012) between highly myopic eyes with CNV and those with no CNV. Ikuno et al. also found choroidal thinning to be more prominent in eyes with myopic CNV compared with non-CNV eyes [20]. The association of choroidal thinning with development of myopic CNV is, as yet, unknown. One hypothesis is that choroidal thinning at the fovea leads to outer retinal hypoxic changes, resulting in release of VEGF—a critical mediator of angiogenesis in the eye [22].

To ensure consistency and to minimize any potential influence from diurnal fluctuation, we measured choroidal thickness between 9:00 a.m. and 12:00 p.m. Studies have shown the choroid to be thickest near midnight and thinnest near noon [23]. Using regression analysis, Margolis and Spaide reported an approximate decrease in choroidal thickness of 15 *μ*m every 10 years [24]. Thus, in order to reduce the influence of age, we chose patients who were 50–70 years old.

Although the primary regulatory role of the choroid in ocular physiology is well known, the in vivo clinical association of choroidal thickness with aqueous PEDF levels is unclear. We examined scatterplots of aqueous PEDF levels and macular choroidal thickness and found positive correlations for the non-CNV (Figure 3), but not the CNV (Figure 4), groups. There was no correlation for the CNV group, because PEDF could not be derived from the atrophic RPE cells in this stage.

Several limitations of our study should be mentioned. First, patients undergoing cataract surgery may differ from patients in general or high myopia patients and, accordingly, it is unclear whether the patients included in our study were representative of normal and high myopia patients. Second, choroidal thickness measurements were performed manually, as there exists no automated device for such measurements. In the present study, two masked readers performed measurements, with open negotiation if there was a difference of >15%. Third, RPE autofluorescence was not evaluated but may be an interesting variable to add to future studies.

In summary, variations in aqueous PEDF levels coincide with changes in macular choroidal thickness in high myopia patients with no CNV, while no such relationship exists in high myopia patients with CNV. Our findings suggest that in vivo macular choroidal thickness may be indicative of aqueous PEDF concentration in high myopia with no CNV, but no indication in high myopia with CNV. Nevertheless, based on previous studies showing that age, refractive error, and/or axial length could significantly influence choroidal thickness, the use of macular choroidal thickness as an indirect assessment of aqueous PEDF concentration might be of limited clinical application. Large-scale studies are recommended, particularly to examine the potential prognostic value of aqueous PEDF levels and choroidal changes.

## Figures and Tables

**Table 1:** Demographic and clinical characteristics.

Variables	Control group (*n* = 42)	High myopia without CNV group (*n* = 36)	High myopia with CNV group (*n* = 14)	*P* value
Mean age (years)	58.5 ± 5.3	59.6 ± 4.9	57.6 ± 5.4	0.573^*∗*^
Axial length (mm)	24.3 ± 0.6	28.5 ± 1.1	29.6 ± 1.3	<0.001^*∗*^
Refractive error (D)	−0.29 ± 1.42	−12.3 ± 4.7	−15.2 ± 3.1	<0.001^*∗*^
Male gender (%)	42.9	44.4	42.9	0.916^*∗∗*^
IOP (mmHg)	14.5 ± 2.8	15.8 ± 2.6	15.5 ± 3.2	0.357^*∗*^

^*∗*^Kruskal-Wallis *H*-test, compared among control, high myopia without CNV (non-CNV), and high myopia with CNV (CNV) groups.

^*∗∗*^Fisher's exact *t*-test compared between control, non-CNV, and CNV groups.

CNV, choroidal neovascularization; D, diopters; IOP, intraocular pressure.
